# Case Report: Calcium sensing receptor gene gain of function mutations: a case series and report of 2 novel mutations

**DOI:** 10.3389/fendo.2023.1215036

**Published:** 2023-08-15

**Authors:** Dalal S. Ali, Francesca Marini, Farah Alsarraf, Hatim Alalwani, Abdulrahman Alamri, Aliya A. Khan, Maria Luisa Brandi

**Affiliations:** ^1^ Division of Endocrinology and Metabolism, McMaster University, Hamilton, ON, Canada; ^2^ Fondazione Italiana Ricerca sulle Malattie dell'Osso (FIRMO) Onlus, Italian Foundation for the Research on Bone Diseases, Florence, Italy; ^3^ Donatello Bone Clinic, Villa Donatello Hospital, Sesto Fiorentino, Italy

**Keywords:** hypoparathyroidism, autosomal dominant hypocalcemia, gain of function mutation, CASR gene, familial hypocalcemic hypercalciuria, ADH1

## Abstract

Autosomal dominant hypocalcemia (ADH1) is a genetic disorder characterized by low serum calcium and low or inappropriately normal levels of parathyroid hormone. The disease is caused by a heterozygous activating mutation of the calcium-sensing receptor (*CaSR*) gene, encoding a G-Protein-coupled cell membrane sensor of extracellular calcium concentration mainly expressed by parathyroid glands, renal tubules, and the brain. ADH1 has been linked to 113 unique germline mutations, of which nearly 96% are missense mutations. There is often a lack of a clear genotype/phenotype correlation in the reported literature. Here, we described a case series of 6 unrelated ADH1 probands, each one bearing a gain-of-function *CaSR* mutation, and two children of one of these cases, matching our identified mutations to the same ones previously reported in the literature, and comparing the clinical and biochemical characteristics, as well as the complication profile. As a result of these genetic and clinical comparisons, we propose that a genotype/phenotype correlation may exist because our cases showed similar presentation, characteristics, and severity, with respect to published cases with the same or similar mutations. We also contend that the severity of the presentation is highly influenced by the specific *CaSR* variant. These findings, however, require further evaluation and assessment with a systematic review.

## 1 Introduction

Autosomal dominant hypocalcemia type 1 (ADH1) (OMIM # 601198; Hypocalcemia, Autosomal Dominant, HYPOC1) is a rare genetic endocrine disorder, characterized by hypocalcemia, hyperphosphatemia, with low or inappropriately normal parathyroid hormone (PTH) level (1–4). It is caused by an inherited or *de novo* heterozygote activating mutation of the calcium-sensing receptor (*CaSR*) gene, located on chromosome 3q13.3–q21 (5). The encoded protein, CaSR, is a G-Protein-coupled cell membrane receptor that is mainly expressed in the parathyroid glands, renal tubules, and the brain (6–9). The CaSR is the key sensor in maintaining extracellular calcium homeostasis, it detects small changes in the serum ionized calcium (iCa^2+^) level and modifies PTH synthesis and release accordingly, to maintain the serum calcium level within the normal range (10–12). In ADH1, the CaSR is activated, even in the presence of normal or low serum calcium levels, resulting in the suppression of PTH synthesis and release, as well as a decrease in renal Ca^+2^ reabsorption (13–15), causing hypocalcemia with relative hypercalciuria (11). As a consequence of the increased risk of hypercalciuria, renal complications are not uncommon in patients with ADH1, namely, nephrocalcinosis, nephrolithiasis as well as renal insufficiency. Moreover, since CaSR can affect the calcium and magnesium handling in the kidneys, also the presence of hypomagnesemia is not uncommon in patients with *CaSR* mutations (16).

ADH1 prevalence was estimated at 3.9 per 100,000 in a recent study of 51,289 subjects from a single US healthcare system (17). Over 100 distinct germline-activating mutations of the *CaSR* gene have been described in association with ADH1 (8, 18). The mode of inheritance is autosomal dominant; however, sporadic mutations have also been reported and the diagnosis can be confirmed by DNA analysis (5, 19, 20).

The clinical presentation of ADH1 is heterogeneous and may vary from mild hypocalcemia which is not diagnosed until late adulthood, to more severe presentations during infancy or childhood with recurrent seizures (16, 21–24). Patients with ADH1 may have the following clinical characteristics: carpopedal spasms, paresthesia, arthralgia, muscle fatigue, and seizures (24, 25). Nephrocalcinosis has been observed in more than 10% of patients with ADH1, with a higher prevalence of intracerebral calcifications accounting for more than 35% of affected cases (23). The clinical implication of basal ganglia calcifications (BGC) in patients with ADH1 is yet to be explored. Existing observational data from individuals diagnosed with chronic hypoparathyroidism has revealed a noteworthy correlation between the presence of BGC and two factors: a low calcium -to-phosphorus ratio and hypocalcemia. These findings suggest that there may be an association between BGC and imbalances in calcium and phosphorus levels in patients with chronic hypoparathyroidism (26, 27). Conventional therapy with calcium and active vitamin D has been reported to be associated with increased hypercalciuria, which may eventually increase the risk of nephrolithiasis, nephrocalcinosis, and renal insufficiency (3, 5, 8, 22, 28). Several other treatment modalities have been used in treating symptomatic patients with ADH1 including thiazide diuretics, recombinant human PTH (1-84), recombinant human PTH (1-34) (3, 29, 30), and most recently the use of calcilytix (negative allosteric modulators of the CaSR), which are currently in phase 2b open-Label, dose-ranging study (31, 32).

In this study, we aimed to evaluate the existence of a possible phenotype-genotype correlation in patients with ADH1 through the clinical comparison of our 6 ADH1 cases with previously described cases bearing the same genetic variants. We also aimed to determine the severity of the disease in relation to any specific *CaSR* mutation variant in our patients compared to previously identified variants.

## 2 Patients and methods

### 2.1 Subjects

Herein, we present a case series of six ADH1 unrelated probands, and two children of one of these cases, attending a tertiary center of excellence in the management of calcium disorders in Canada. We obtained informed consent from either the adult participants or, in the case of children, their parents.

### 2.2 Biochemical parameters

The laboratory measurements of various serum parameters were conducted following the standards set by Canadian laboratories. These parameters include ionized calcium, calcium corrected for albumin, phosphorus, magnesium, creatinine, estimated glomerular filtration rate (eGFR), 25 hydroxyvitamin D [25(OH)D3], parathyroid hormone (PTH), and alkaline phosphatase (ALP). For the conversion of Calcium, PTH and 25(OH)D_3_, the following rule may be applied:

- To convert calcium from mmol/L to mg/dL, multiply by 4. The normal range for calcium (Ca^+2^) is 8.6 to 10.3 mg/dL (33).
- To convert PTH from pmol/L to pg/mL, multiply by 10. The normal range for intact PTH is 10-65 pg/mL (33).
- To convert 25(OH)D_3_ from nmol/L to ng/mL, multiply by 0.4. The normal range for 25(OH)D_3_ is 30-50 ng/mL (33).

Data of retrospective laboratory parameters were retrieved from the electronic medical records (EMR).

### 2.3 Imaging

Images to assess for long-term complications of hypoparathyroidism consisted of renal ultrasound (US) and computed tomography scan (CT) of the brain were both retrieved from the EMR. For most of our cases, these investigations were done as part of their routine clinical care.

### 2.4 Genetic analysis

Genomic DNA was extracted by peripheral blood leukocytes, enriched for targeted gene regions using a hybridization-based protocol, and, then, analyzed by Next Generation Sequencing (NGS) technologies to cover the coding regions of the targeted genes plus 10 bases of non-coding DNA flanking each exon, using Illumina’s Reversible Dye Terminator (RDT) platform NovaSeq 6000 with 150 by 150 bp paired-end reads (Illumina, San Diego, CA, USA). Regions with insufficient coverage by NGS were covered by Sanger sequencing. Sanger sequencing was performed following Polymerase Chain Reaction (PCR) amplifications of coding exons and flanking splicing sequences. After purification of the PCR products, cycle sequencing was carried out using the Applied Biosystems incorporated (ABI) Big Dye Terminator v.3.1 kit. Genome build hg19, GRCh37 (Feb 2009) was used as a reference for all cases.

### 2.5 Literature search for CaSR variants

We searched PubMed, Google Scholar, and MEDLINE from 1994 to November 2022 using the following keywords: Hypoparathyroidism, autosomal dominant hypocalcemia, gain of function mutation, *CaSR* gene, hypercalciuric hypocalcemia, ADH1, to find published cases reporting the same *CaSR* variants we identified in our ADH1 cases.

We also searched some of the most common databases of human gene variants, including OMIM, ClinVar, Human Gene Mutation Database (HGMD), and GnomAD to look for the same variants identified in our ADH1 cases.

## 3 Results

The clinical presentations and characteristics of our ADH1 patients are summarized in **Table 1**. Of our cases, 37.5% are of a confirmed familial cause (cases 1, 7, and 8), and 25% are highly likely secondary to *de novo* mutation due to a lack of family history of hypocalcemia or parathyroid disorder (cases 4 and 5). The remaining 37.5% are cases with a strong family history of hypocalcemia/hypoparathyroidism but with no confirmed molecular diagnosis, which we can infer are familial, making 75% of our cases familial. The lack of molecular diagnosis in family members of our cohort is multifactorial and predominantly related to access issues or living in a different part of the world. All of our mutation variants are heterozygous. **Table 2** summarizes our identified *CaSR* variants, and their comparison with previously published cases.

**Table 1:** Clinical characteristics of the studied patients with ADH1.

ID	Age (yrs)	BMI (Kg/m2)	Ethnicity	Sex	TTD (yrs)	Biochemical profile			Renal and neurological complications	Associated features	FHx	Genetic mutation
						Ca^2+^	PTH	PO_4_				
1	25	44	Caucasian	F	1.5	Low	Low	High	Seizures BG calcifications Hypercalciuria	Hypomagnesemia short 4^th^, 5^th^ metacarpals Café-au-lait spots	Father: HypoPT * 2 children (ADH1)	c.2363T>G; p.Phe788Cys
2	24	20.6	NA	M	20	Low	Low-N	High	Nephrolithiasis Intermittent Hypercalciuria	VUS in the *FAM111A* gene	Mother: idiopathic HypoPT*	c.571G>A; p.Glu191Lys
3	41	36.4	Caucasian	F	34	Low	N	N			Father: Hypocalcemia *	c.377A>T; p.Asp126Val
4	32	27.5	Caucasian	M	10 (days)	Low	Low	High (N after PTH Rx)	Seizures Nephrocalcinosis Nephrolithiasis CKD stage 3 Hypercalciuria	Hypokalemia Hypomagnesemia	No	c.2482A>C; p.T828P
5	39	21	South Asian	F	37	Low	N	N	Seizures age 31 Nephrolithiasis		No	c.2468T>A; p.lle823Asn
6	47	24.5	Caucasian	F	42	Low	N	N	Intermittent Hypercalciuria		Maternal grandmother: Hypocalcemia *	c.1756G>A; p.Glu586Lys
7 Child #1 of 1	5	18.3 (95%tile)	Caucasian	F	2 wks	Low	Low	High	Bilateral nephrocalcinosis, Nephrolithiasis Hypercalciuria	Hypomagnesemia	Mother, maternal grandfather	c.2363T>G; p.Phe788Cys
8 Child #2 of 1	14 (mo)	19.8 (98%tile)	Caucasian	M	2 wks	Low	Low	Low/High	Seizures Nephrocalcinosis	Hypomagnesemia	Mother, maternal grandfather	c.2363T>G; p.Phe788Cys
**% of occurrence in all cases**						**100%** **Low** **Ca^2+^**	**63%** **Low** **PTH**	**63%** **High** **PO_4_**	**63% Hypercalciuria** **50% Seizures** **50% Nephrolithiasis** **37.5** **% Nephrocalcinosis** **13% BG calcifications**	**50% Hypomagnesemia** **13% Hypokalemia**	**75% with positive family history**	

TTD, Time to diagnosis; BMI, Body mass index; PTH, parathyroid hormone; BG, basal ganglia; FHx, family history; CKD, chronic kidney disease; Ca^+2^, calcium; PO_4_, phosphorus; N, within the normal range; HypoPT, hypoparathyroidism; mo, months; wks, weeks; %tile, percentile based on WHO (Girls 2-6 years and Boys, 0-2 years) BMI-for-age; * represents no molecular diagnosis of ADH1; %, percentage; NA, not available; F, female; M, male; Bold, cumulative findings from all the cases.

**Table 2:** Genotype-phenotype correlation, with respect to previously published cases.

Cases and citation	Mutation of the CaSR gene	Chldhood presentation	No. Of cases	Renal Complications	Basal Ganglia Calcifications	Seizure at diagnosis	Hypercalciuria	Biochemical profile	FHx	Associated features
Case 1	c.2363T>G; p.Phe788Cys	+	3	NC (2/3) NL (1/3)	+ (1/3)	+	+	↓Ca ↓ Mg ↓PTH ↑PO4	+	Short fingers Café-au-lait spots 1/3
Watanabe T et al. (21)		Yes (2/3) Mother – retrospective	3	NR	+ age 12 + age 16 2/3	+ (2/3)	+	↓Ca ↓ Mg ↓PTH ↑PO4	+	
Lienhardt A et al. (22)		Yes	1	NR	NR	NR	NR	↓Ca ↓ Mg NPTH	*de novo*	
Kinoshita Y et al. (16)		Yes	1	NR	NR	NR	NR	↓Ca ↓ Mg ↓PTH ↑PO4	*de novo*	
Elston et al. (34)		Yes	1	NC	NR	+	+	↓Ca ↓ Mg ↓PTH ↑PO4	Likely *de novo* Parents rejected DNA testing (both Ca normal)	myoclonic jerks of upper limbs
Mora et al. (35)		Yes	1	NC	+	+	+	↓Ca ↓ Mg ↓PTH ↑PO4	*de novo*	
A. García-Castaño et al. (36)		No	1	NL	+	NR	+	↓Ca ↓ Mg ↓PTH ↑PO4	*de novo*	
Kamiyoshi et al. (37)	c.2326 T> G; p.Phe788Cys	Yes (1/2)	2	NC (1/2)	NR	NR	NR	↓Ca ↓ Mg ↓PTH	+	↓K, metabolic alkalosis, short stature 1/2
**Occurrence among cases**		**77%**	**N=13**	**38.5% NC** **15% NL**	**38.5%**	**54%**	**70%**	**100% ↓ Mg 77% ↑PO4**	**61.5% AD** **38.5% *de novo***	
Case 2	c.571G>A; p.Glu191Lys	No	1	NL	–	No	Intermittent	↓Ca ↓PTH ↑PO4	+	
Pearce SHS et al. (5)		No (3/4)	4	NC/RI (3/4)	–	No	+ (post-treatment) (2/4)	↓Ca ↓PTH ↑PO4 N PTH (↓ after Rx with calcitriol)	+	
**Occurrence among cases**		**80%**	**N=5**	**80%**			**60%**		**100% AD**	
Case 3	c.377A>T; p.Asp126Val	No	1	–	–	–	–	↓ Ca, NPTH, NPO4	+	
Rasmussen AQ et al. (38)		No	3	NC (2/3)	–	–	–	↓Ca, ↓/N PTH, ↑/N PO4	+	severe muscle pain, arthralgia, tetany, abdominal pain, and fatigue
**Occurrence among cases**		**100%**	**N=4**	**50%**					**100% AD**	
Case 4	c.2482A>C; Possible Novel Mutation p.T828P.									
Case 5	c.2468T>A; p.lle823Asn	Novel Mutation								
Case 6	c.1756G>A; p.Glu586Lys	Novel Mutation								

NC, nephrocalcinosis; NL, nephrolithiasis; NR, not reported; RI, renal impairement; AD, autosomal dominant; N, normal; ↓Ca, low serum calcium; ↓ Mg, low serum magnesium; ↓PTH, low parathyroid hormone; ↑PO4, high serum phosphorus; ↓K, low potassium; +, present; -, absent; Bold, The collective results, expressed as a percentage, among individuals who bear the same mutation variant.

Our six unrelated ADH1 probands showed distinct clinical presentations, while the three ADH1 cases within the same pedigree (cases 1, 7, and 8) shared similar presentation and clinical characteristics, see **Table 1**. The biochemical profile differed amongst the cases in terms of the degree of hypocalcemia, associated hypercalciuria, or other biochemical abnormalities, such as hypomagnesemia and hypokalemia, as well as response to therapy. Individualized biochemical profiles are summarized in **Table 3**.

**Table 3:** Biochemical profiles of the studied 8 cases prior to and after therapy.

Biochemical Parameter	Corrected Ca^+2^		Phosphorus		PTH		Magnesium		25(OH)D_3_		24 hr urine Ca^+2^		1,25(OH)_2_D_3_	Ca^2+^/PO4 ratio
	Pre Rx	Mean post Rx	Pre Rx	Mean post Rx	Pre Rx	Mean post Rx	Pre Rx	Mean post Rx	Pre Rx	Mean post Rx	Pre Rx	Mean post Rx		
Normal Range	2.15-2.6 mmol/L*		0.8-1.45 mmol/L		1.6-6.9 pmol/L**		0.65-1.05 mmol/L		75-125 nmol/L***		2.5-7.5 mmol/day		64-226 pmol/L	
Case 1^⊗^	NA	1.9±0.25	NA	2.1±0.22	<0.6	<0.6	NA	0.61±0.11	NA	74±17	NA	NA	163	0.904
Case 2^¥^	1.73	1.7±0.12	1.74	1.8±0.10	1.7	1.4±0.1	0.78	0.73±0.03	53	63±23	2.2	6±2.2	69	0.944
Case 3^¥^	1.78	1.9±0.11	1.81	1.3±0.42	1.5	1.8±1.8	0.65	0.82±0.11	76	78±21	NA	3.7±2.7	103	1.46
Case 4˄	NA	2.1±0.40	NA	1.6±0.14	<0.3	<0.3	NA	0.81±0.11	NA	76±66	NA	4.1±2.0	198	1.31
Case 5^¥^	2.14	2.0±0.02	1.46	1.45±0.1	3.8	3.0±0.7	0.96	0.85±0.02	70	51±15	3.25	4.7±2.2	141	1.37
Case 6^¥^	NA	2.1±0.11	NA	1.1±0.24	4.6	1.4±0.2	NA	0.75±0.07	NA	79±7	NA	8.0±3.0	124	1.9
Normal range (peds)	2.2-2.7 mmol/L		1.55 -2.65 mmol/L		1.4-6.8 pmol/L		0.65-1.05 mmol/L							
Child 1 of case 1^‡^	NA	2.1±0.11	1.4	2.8 ±0.2	<0.6	<0.6	0.50	0.67±0.02	61	72±16	2.7			0.75
Child 2 of case 1^‡^	1.8	2.3±0.02	0.8	2.6±0.3	<0.4	<0.4	0.44	0.66±0.1	157	134±32	3.2		118	0.88

Explanation: values are presented as mean±SD.

NA, not availabe; PTH, parathyroid hormone; Ca^+2^, calcium; 25(OH)D_3_, 25 hydroxyvitamin D; Rx, treatment; Peds, pediatrics; 1,25(OH)_2_D_3_, 1,25 dihydroxyvitamin D.

* to convert serum Ca^+2^ from mmol/L to mg/dL multiply by 4 (normal Ca^+2^ range: 8.6 to 10.3 mg/dL) (33).

** to convert PTH from pmol/L to pg/mL multiply by 10 (normal intact PTH range 10-65 pg/mL) (33).

*** to convert 25(OH)D_3_ from nmol/L to ng/ml multiply by 0.4 (normal 25(OH)D_3_ range 30-50 ng/mL) (33).

⊗ on calcium, calcitriol, magnesium, and cholecalciferol.

¥ on calcium, calcitriol, and cholecalciferol.

˄ on PTH therapy (trial medication) (39), magnesium and cholecalciferol.

‡ on calcium, calcitriol, magnesium, and hydrochlorothiazide.

### 3.1 Case 1

A 25-year-old woman with ADH1, diagnosed at the age of 17 months after presenting with grand mal seizure and found to have hypocalcemia. She has an activating mutation of the *CaSR* gene (c.2363T>G; p. Phe788Cys). Replacement with calcium and active vitamin D was initiated at the time of diagnosis with the goal of maintaining the serum-corrected calcium in the low or just below the normal reference range. Her father had severe hypocalcemia and hypomagnesemia, as well as Fahr disease, and suffered from cognitive deterioration over time, necessitating institutionalization. Fahr disease, also known as familial idiopathic basal ganglia calcification, is a rare inherited neurological disorder that is characterized by abnormal deposition of calcium in the basal ganglia, thalamus, and cerebral cortex and was first described by Karl Theodor Fahr in Germany in 1930 (40, 41). She has two other siblings, none of whom have ADH1. However, both of her children have confirmed clinical and molecular diagnoses of ADH1. On physical examination, she is noted to have short 4^th^ and 5^th^ metacarpals, café-au-lait spots on the right forearm and back, and had no dysmorphic features. She has bilateral basal ganglia calcifications as well as intracranial calcifications on imaging, diagnosed at the age of 11 years, but no evidence of neurological or cognitive impairment to date. There is no evidence of nephrocalcinosis on renal ultrasound. Despite requiring high doses of calcium and active vitamin D supplements, the serum corrected calcium remains below the normal reference range at approximately 1.8-1.9 mmol/L (NR: 2.15-2.6), with hyperphosphatemia, hypercalciuria, and multiple hospitalizations for hypocalcemia. On this basis, our patient was offered PTH therapy on several occasions, however, this was deferred due to family planning. She has currently completed her family and is being considered for calcilytix therapy. Her most recent treatment regimen consisted of calcitriol 1.50 mcg twice daily, calcium carbonate 3000 mg twice daily, magnesium oxide 1500 mg twice daily, and cholecalciferol 2000 IU daily. The biochemical profile is summarized in **Table 3**.

#### 3.1.1 Case 1 Pregnancies

This patient had 2 pregnancies, and, in both pregnancies, she required multiple hospital admissions due to hypocalcemia and non-adherence concerns. Her requirements of calcium and active vitamin D tripled in pregnancy especially in the 2^nd^ trimester in both pregnancies with subsequent declines in the requirements during lactation. Both of her babies were born preterm at 37- and 34-weeks’ gestation, respectively; and required NICU admission for 2 weeks for the management of hypocalcemia. Both children have ADH1 and bear the same mutation variant as the mother, c.2363T>G; p.Phe788Cys. Child 1 is currently 5 years old, has normal milestones to date, and is maintained on calcium carbonate (500 mg three times daily), calcitriol (1.6 mcg twice daily), magnesium glucoheptonate (400 mg three times daily), potassium citrate (25 mg daily), as well as hydrochlorothiazide (5 mg twice daily). With regards to complications, nephrocalcinosis, nephrolithiasis, and recurrent urinary tract infection are present. Child 2 is 14 months old. The mother had a late preterm birth via emergency c-section due to decreased fetal movements and a low biophysical profile (BPP) of 2/8. She also had mild polyhydramnios. Following birth, the infant received APGAR scores of 3, 7, and 8, necessitating suctioning and subsequent continuous positive airway pressure therapy (CPAP). Alongside these challenges, an atrial septal defect (ASD) was detected. The infant also experienced hypocalcemic seizures and underwent treatment involving calcium carbonate (70 mg elemental calcium three times daily), calcitriol (1 mcg twice daily), magnesium glucoheptonate (880 mg 6 times daily), and hydrochlorothiazide (5 mg twice daily). Notably, initial imaging did not reveal renal calcifications, however, repeated renal ultrasound has shown evidence of bilateral nephrocalcinosis. Nephrolithiasis, and basal ganglia calcifications have not been documented.

### 3.2 Case 2

A 24-year-old man was diagnosed with ADH1 at the age of 20 years, after presenting with fatigue, muscle cramping, and difficulty in concentration. He has a heterozygous pathogenic activating variant of the *CaSR* gene (c.571G>A; p.Glu191Lys), and a variant of unknown significance (VUS) in the *FAM111A* gene (c.228del; p.Thr77Glnfs*6). Mutation in the *FAM111A* gene has been linked to Kenny-Caffey syndrome (KCS), which is a genetic disorder characterized by short stature and impaired skeletal development as well as hypoparathyroidism (42). In terms of complications, he developed nephrolithiasis and has had a history of fragility fracture. His mother has idiopathic hypoparathyroidism diagnosed at age 27 years with symptomatic hypocalcemia, mainly paresthesia and muscle cramps, and is currently well-maintained on calcium and active vitamin D supplements. On examination, our patient does not show any features that suggest other inherited or syndromic causes of hypoparathyroidism including, but not limited to, autoimmune polyendocrinopathy syndrome type I (APS1), Barakat syndrome, or DiGeorge syndrome. He is currently maintained on calcium carbonate 2000 mg daily with meals, calcitriol 0.25 mcg daily, and cholecalciferol 1000 IU weekly. The biochemical profile is summarized in **Table 3**.

### 3.3 Case 3

A 41-year-old woman has ADH1 with a confirmed activating mutation in the *CaSR* gene (c.377A>T, p.Asp126Val). She also has a VUS mutation in the *AIRE* gene (c.1322C>T, p.Thr441Met), a gene whose homozygous, compound heterozygous, or heterozygous mutations have been associated with APS1 with or without reversible metaphyseal dysplasia. She initially presented with numbness and tingling involving the face, hands and feet, leg cramps, and bronchospasm at the age of 34 years. There were no clinical or biochemical features to suggest APS1. She had a history of oral and vaginal candidiasis which has resolved, and the adrenal and gonadal functions have been normal. There was no evidence of basal ganglia calcifications, nephrocalcinosis, or nephrolithiasis on imaging. The patient’s father had a history of hypocalcemia and was on calcium supplements throughout his life; he also had chronic kidney disease. Similarly, both of her paternal uncles and three paternal cousins suffer from hypocalcemia. However, her two sisters remain healthy and have not experienced any episodes of hypocalcemia. Genetic testing is not available for her family members. On physical examination she has hypertension, and blood pressure is maintained on antihypertensive medications. There are no dysmorphic features. She is currently maintained on calcium carbonate 5000 mg daily with meals, calcitriol 0.5 mcg twice daily, and cholecalciferol 6000 IU daily. The biochemical profile is summarized in **Table 3**.

#### 3.3.1 Case 3 Pregnancies

She was pregnant a total of 5 times, 2 of which resulted in miscarriages at 14 weeks gestation, and the remaining 3 pregnancies were complicated by preeclampsia, resulting in preterm delivery. She denied any seizure or muscle twitching in any of her children, none required ventilatory support and all children have normal milestones.

### 3.4 Case 4

A 32-year-old man with ADH1 was diagnosed at the age of 10 days after presenting with a generalized seizure. He has an activating mutation of the *CaSR* gene (c.2482A>C; p.Thr828Pro), he also has associated hypomagnesemia, hypokalemia, and hypercalciuria. He has a wide fluctuation in serum calcium with multiple hospital admissions with severe hypocalcemia complicated by seizures. He also has a history of recurrent kidney stones from the age of 8 years requiring lithotripsy, the last stone was passed at the age of 31 years. Moreover, he has chronic kidney disease stage 3. No family history of note, his mother has post-surgical hypoparathyroidism, and his father and sister have normal calcium levels. He has no dysmorphic features, and his other system examination is unremarkable. He is currently enrolled in phase 3 TransCon PTH (TC PTH) trial; TC PTH is a prodrug of PTH (1-34) that is transiently conjugated to polyethylene glycol resulting in stability of the PTH level (39). During his early 20s, he was on thiazide diuretics in addition to conventional therapy with calcium and calcitriol. He is now maintained on Potassium 500 mg daily, Magnesium Rougier 15ml once a week, cholecalciferol 3000 IU daily, TC PTH: 60 mcg daily (trial medication). The biochemical profile is summarized in **Table 3**.

### 3.5 Case 5

A 39-year-old woman has ADH1 with biochemical evidence of low serum calcium and inappropriately normal PTH level and a confirmed heterozygous VUS mutation of the *CaSR* gene (c.2468T>A,p.lle823Asn). She also has a VUS mutation in the *HADHA* gene (c.80G>T, p.Arg27Leu), a gene whose homozygous or compound heterozygous mutation has been associated with mitochondrial trifunctional protein deficiency (MTPD). Hypocalcemia was discovered in a the lab profile, along with hypocalcemic symptoms, predominantly paresthesia, for two years prior to presentation at the age of 37. This *CaSR* genetic variant is a novel mutation that has not previously been reported in the literature or mutation databases. The patient has not had previous history of radiation therapy to the neck, nor other syndromic features of inherited hypoparathyroidism disorders. She had one episode of seizure at the age of 30 years at which time the serum calcium level was not reported. She also had kidney stones, the last of which was passed at the age of 38 years. Renal ultrasound following the passage of the kidney stone showed preserved corticomedullary differentiation bilaterally. There were no basal ganglia calcifications on the brain CT scan. She has no family history of hypocalcemia in both of her parents and siblings. She has one aunt who had a seizure disorder, but the serum calcium level is unknown. The hypocalcemia in her case is mild and is managed with multivitamins (calcium 130mg), calcitriol 0.25 mcg 3x/week, and ergocalciferol 50,000 IU weekly. The lab profile is summarized in **Table 3**.

#### 3.5.1 Case 5 Pregnancy 

She has one child, born at 40 weeks gestation and breastfed for 8 months with no issues, and has normal milestones to date. No miscarriages.

### 3.6 Case 6

A 47-year-old woman with a molecular diagnosis of ADH1. She has a VUS heterozygous mutation of the *CaSR* gene, c.1756G>A; p.Glu586Lys. She was first diagnosed with hypocalcemia at the age of 44 years after experiencing numbness and tingling in her face and hands. Her mother and maternal grandmother both had hypocalcemia but have not obtained a molecular diagnosis. Hypercalciuria is present, the renal ultrasound did not show evidence of kidney stones or nephrocalcinosis and the brain MRI did not show evidence of basal ganglia calcifications. She is currently maintained on calcium carbonate powder 600 mg daily, calcitriol 0.25 mcg daily, and cholecalciferol 1000 IU daily.

## 4 Discussion

In patients with ADH1 (5), there is an established correlation between the presence of an activating mutation in the *CaSR* gene and the presence of hypocalcemia. However, the literature is lacking in identifying other clinical areas of phenotype-genotype correlation in ADH1 patients (5). In our ADH1 case series, we observed a potential correlation between the presence of specific *CaSR* variants, affecting specific domains of the CaSR protein, and the clinical characteristics of the patients. In addition, to better evaluate possible association between *CaSR* variants and ADH1 clinical manifestations, we also clinically compared our cases with the ones, published to date, bearing the same or similar *CaSR* variants, to identify the presence of common clinical characteristics.

The degree of hypocalcemia and clinical presentation in patients with activating mutation of the *CaSR* gene have been previously evaluated (18), showing that there is no association between values of serum calcium with the three different mutation sites in the CaSR protein (ligand-binding extracellular domains, 7 transmembrane domains, or C-terminus intracellular domain) (18). However, in a recent systematic review, it was noted that patients with more severe disease had lower serum calcium levels in comparison to asymptomatic patients (7.6 ± 0.7 mg/dL; p<.0001) or moderately symptomatic (7.4 ± 0.5 mg/dL;p< 0.01) patients with ADH1 (8). In our cohort, Case 2, bearing the p.Glu191Lys variant in the ligand-binding 2 extracellular domain, had the lowest mean value of serum-corrected calcium but did not show a severe clinical presentation, in terms of the presence of seizures, renal impairment, recurrent hospital admissions with hypocalcemia and requirement of frequent dose adjustments (43). By contrast, in our case series, we observed a correlation between the severity of hyperphosphatemia and the occurrence of complications affecting both the renal and neurological systems. However, it is important to note that this study is not powered to establish a causal relationship.

The search on the database of human mutations and Pubmed allowed us to identify a total of 10 published cases, in addition to our 3 cases, bearing the p.Phe788Cys mutation. This mutation is located in the fifth transmembrane (TM5) domain encoded by exon 7 of the *CaSR* gene. All but one case manifested the disease in early childhood, with two undisclosed times of diagnosis, accounting for 77% of the cases presenting in early childhood. The patient with the pPhe788Cys variant who had an adult presentation of ADH1 also bore another mutation in the *CaSR* gene, thought to be an activating mutation, which may have influenced the clinical presentation and made it less prevalent (36). Moreover, the neurological involvement, such as hypocalcemic seizures and basal ganglia calcifications was more prevalent in patients carrying this mutation compared to others. Seizures were reported at diagnosis in 7 of the 13 cases with the p.Phe788Cys mutation, accounting for 54% of the cases; the remaining 6 cases did not indicate or deny the presence of seizures. Basal ganglia calcification was reported in 5 out of the 13 cases (38.5%). Nephrolithiasis was present in 15% (2/13 cases) and nephrocalcinosis was present in 38.5% of the cases (5/13). Patients with this mutation shared the presence of at least three complications associated with ADH1, which could be a marker of severity. Although family history was positive in three pedigrees, accounting for 61.5% being of familial inheritance, the remaining 38.5% were confirmed to be of *de novo* inheritance. In our three genetically related cases with the p.Phe788Cys variant (cases 1, 7, and 8), seizures were described in two cases (66.7%), while basal ganglia calcifications were reported to date only in the 25-year-old mother.

It was previously reported that activating mutations in the fifth transmembrane (TM5) domain of the CaSR protein cause severe familial hypoparathyroidism (21). This is also evident in our three patients bearing the p.Phe788Cys variant, as well as in published cases with the same mutation. The individuals with this mutation, including our cases, shared a unique feature consisting in the fact that calcium levels had to be maintained within the range of 1.75- 1.9 mmol/L (7–7.6 mg/dL) in order to avoid symptoms and complications similar to those experienced by patients with frank hypercalcemia. Mutations in the TM5 domain render the CaSR protein more sensitive than normal to activation by Ca^+2^. A previous report documented another mutation at the codon 788, the p.Phe788Leu, in two individuals who had ADH1 with basal ganglia calcifications and nephrocalcinosis (44). Both these missense mutations involving the codon 788 demonstrated a leftward shift in the concentration-response curve in relation to extracellular calcium change and intracellular calcium increase (21, 44).

We identified in one ADH1 case the p.Thr828Pro variant that has not previously been reported, despite the fact that three publications were listed on ClinVar, none of which reported this specific mutation in the manuscript (16, 23, 45). Another missense mutation (p.Thr828Asn), affecting the same codon, has been described as associated with ADH1, however, the published study did not offer additional clinical information (9). Therefore, it could be inferred that alteration at codon 828 may result in an activating mutation of the *CaSR* gene leading to the development of ADH1. Interestingly, our p.Thr828Pro-bearing patient (case 4) shared similar features to the three patients with the p.Phe788Cys variant (cases 1, 7, and 8), in which the disease presentation is early in life (10 days old), there are recurrent seizures and multiple hospital admissions with hypocalcemia, as well as the presence of nephrocalcinosis and renal insufficiency. Moreover, hypomagnesemia was present in all these four cases.

Time to ADH1 diagnosis was variable in our eight ADH1 cases, presumably reflecting the severity of the disease. Indeed, more severe cases tend to declare themselves early in life, as for our cases 1, 4, 7, and 8, while milder forms tend to present later in life with mild symptoms. Both the p.Phe788Cys and the p.Thr828Pro mutations are located in the exon 7-encoded TM5 domain of the CaSR protein. This suggests that the alteration of this domain could be causative for the shared clinical characteristics in our 4 ADH1 cases and for the earlier presentation of the clinical phenotype. In addition, another missense variant (p.Leu773Arg) located at the TM5 domain, was associated with an ADH1 clinical phenotype characterized by an early onset hypocalcemic seizure, hyperphosphatemia, and basal ganglia calcifications (46), strengthening the hypothesis that single amino acid substitutions at the TM5 domain could be predictive of an early onset disease with neurological involvement.

By contrast, another novel missense mutation (p.Met734Thr) in the TM5 region was described, from a German survey, in an asymptomatic patient with hypocalcemia and low PTH level. However, this mutation lacked further familial and functional characterizations to be classified as “damaging or pathogenic” and was believed to be benign (23).

Based on data from our patients and results from the published studies, we hypothesize that mutations affecting the TM5 domain of the CaSR protein could be responsible for a more severe form of the disease, and they may be associated with a higher rate of neurological manifestations, such as a history of hypocalcemic seizures and the presence of basal ganglia calcifications, as well as of renal involvement, including nephrocalcinosis, nephrolithiasis, and renal insufficiency.

In this study, we also described two novel mutations of the *CaSR* gene: c.2468T>A; p.Ile823Asn and c.1756G>A; p.Glu586Lys. Neither of these variants has been reported in ClinVar, Human Gene Mutation Database (HGMD), GnomAD, or PubMed. In silico analysis of these two missense mutations with PolyPhen-2 (a tool for the prediction of the functional effect of a non-synonymous single amino acid substitution on a human protein) predicted the Ile to Asn substitution at position 823 as “probably damaging” with a score of 1.000 (sensitivity: 0.00; specificity: 1.00) and the Glu to Lys substitution at position 586 as “benign” with a score of 0.095 (sensitivity: 0.93; specificity: 0.85). Interestingly, another *CaSR* variant affecting the same codon 586 (c.1758G>C, p.Glu586Asp) has been previously reported, by a single entry on ClinVar, as a VUS variant for the familial hypocalciuric hypercalcemia type 1 (HHC1); a clinical phenotype, mirroring the ADH1, characterized by a lifelong elevation of serum calcium concentrations, an inappropriately low urinary calcium excretion and a normal or mildly elevated PTH, and caused by heterozygous loss-of-function mutations in the *CaSR* gene. However, there were no citations or functional evidence of this mutation.

In a recent systematic review, it was found that 93% of patients with ADH1 had an incomplete diagnosis of idiopathic hypoparathyroidism or hypocalcemia at the time of presentation, and 7% of the cases were misdiagnosed with seizures or respiratory/cardiovascular disorders (8). This is reflected in our cohort, as 37.5% of our patients’ family members were diagnosed with idiopathic hypoparathyroidism and hypocalcemia.

In conclusion, our data and the comparison with previously published ADH1 cases suggested the existence of a genotype/phenotype correlation regarding the presence of specific ADH1-related clinical manifestations and the early onset and/or severity of the disease. These findings, however, require further evaluation and assessment with a systematic review.

## Data availability statement

The original contributions presented in the study are included in the article. Further inquiries can be directed to the corresponding author.

## Ethics statement

Ethical review and approval was obtained. This study uses subjects enrolled to the CALIBRATE study: a Phase 3, Randomized, Open-Label Study that evaluated the efficacy and safety of Encaleret compared to the standard of care in participants with ADH1 (NCT05680818). Written informed consent was obtained from the individual(s), and minor(s)’ legal guardian/next of kin, for the publication of any potentially identifiable images or data included in this article.

## Author contributions

Design and conceptualization of the project: DA, AK, MB. Analysis and interpretation of patients’ clinical and biochemical data: DA, FM. Review/editing of the manuscript: DA, FM, FA, HA, AA, AK, MB. All authors contributed to the article and approved the submitted version.
